# The Cell Protective Effect of Adenine on Hypoxia–Reoxygenation Injury through PPAR Delta Activation

**DOI:** 10.3390/life11121408

**Published:** 2021-12-16

**Authors:** Jyh-Gang Leu, Chien-Mei Wang, Chao-Yi Chen, Yi-Feng Yang, Chin-Yu Shih, Jiun-Tsai Lin, Han-Min Chen, Yao-Jen Liang

**Affiliations:** 1 School of Medicine, Fu-Jen Catholic University, New Taipei City 24205, Taiwan; m004224@ms.skh.org.tw; 2 Division of Nephrology, Department of Internal Medicine, Shin Kong Wu Ho-Su Memorial Hospital, Taipei 11101, Taiwan; 3 Department and Institute of Life Science, Fu-Jen Catholic University, New Taipei City 24205, Taiwan; may.cm.wang@cymmetrik.com (C.-M.W.); 056489@mail.fju.edu.tw (H.-M.C.); 4 Graduate Institute of Applied Science and Engineering, Fu-Jen Catholic University, New Taipei City 24205, Taiwan; 401068029@mail.fju.edu.tw (C.-Y.C.); 403068073@mail.fju.edu.tw (Y.-F.Y.); 407068019@mail.fju.edu.tw (C.-Y.S.); 5 Energenesis Biomedical Co., Ltd., Taipei 11492, Taiwan; bio@energenesis-biomedical.com

**Keywords:** adenine, ischemia-reperfusion, myocardial infarction, antioxidation, cell cycle

## Abstract

Ischemia followed by blood supply reperfusion in cardiomyocytes leads to an overproduction of free radicals and a rapid decrease of adenosine triphosphate concentration. The cardioprotective effect of a potential drug, adenine, was evaluated using H9c2 rat cardiomyoblasts. After hypoxia–reoxygenation (HR) treatment consisting of hypoxia for 21 h followed by reoxygenation for 6 h, it was revealed that pretreatment with 200 µM adenine for 2 h effectively prevented HR-induced cell death. Adenine also significantly decreased the production of reactive oxygen species and reduced cell apoptosis after HR injury. The antioxidant effect of adenine was also revealed in this study. Adenine pretreatment significantly reduced the expression of activating transcription factor 4 (ATF4) and glucose-regulated protein 78 (GRP78) proteins, and protein disulfide isomerase induced a protective effect on mitochondria after HR stimulation. Intracellular adenosine monophosphate-activated protein kinase, peroxisome proliferator-activated receptor delta (PPARδ), and perilipin levels were increased by adenine after HR stimulation. Adenine had a protective effect in HR-damaged H9c2 cells. It may be used in multiple preventive medicines in the future.

## 1. Introduction

Coronary artery disease is a major cause of mortality and disability in most countries in the world. Obstruction of coronary artery results in ischemic myocardial injury and permanent cell death [1]. Reperfusion therapy with thrombolytic drugs or percutaneous coronary intervention are considered the most effective therapeutic strategies for myocardial ischemia [2]. Compared to permanent coronary artery obstruction, however, reperfusion of blood flow after reperfusion therapy causes additional cell damage and death after ischemia, referred to as myocardial ischemia-reperfusion injury [3].

The hypoxic conditions during ischemia result in depletion of ATP and lowering of intracellular pH. With reperfusion, oxygen becomes rapidly available, allowing oxidative phosphorylation to resume. This switch back to aerobic metabolism generates reactive oxygen species, with levels remaining elevated for several hours [4,5]. The increased oxidative stress coupled with pH restoration induce the opening of the mitochondrial permeability transition pore [6]. This large, nonspecific pore allows passage between the mitochondrial matrix and the cytosol, which disrupts the normally finely regulated transport of protons and ions and leads to swelling and rupture of the mitochondria, which triggers necrotic cell death [7].

Adenine is a kind of purine. Intracellular adenine may react with phosphoribosyl pyrophosphate (PRPP) to produce adenosine monophosphate (AMP) through the catalysis of adenine phosphoribosyltransferase (APRT), an important enzyme in the salvage pathway [8]. A rise of intracellular AMP:ATP ratio causes phosphorylation and activation of AMP-activated protein kinase (AMPK), a key sensor of energy homeostasis in eukaryotes [9]. Previous studies reported that AMPK was involved in modulating cellular inflammatory response [10]. Activated AMPK represses NF-κB translocation, the expression of NF-κB target genes, and monocyte adhesion to endothelial cells [11]. Numerous studies also supported the anti-inflammatory effect of AMPK activators in response to proinflammatory stimuli [12]. 

Adenine was shown to induce the phosphorylation of AMPK in both a time- and dose-dependent manner as well as its downstream target acetyl Co-A carboxylase (ACC). Adenine also attenuated NF-κB targeting of gene expression in a dose-dependent manner and decreased monocyte adhesion to HUVECs following tumor necrosis factor (TNF-α) treatment. The anti-inflammatory role of adenine is mediated by AMPK. The role of adenine as an AMPK activator is mediated by APRT catabolism. Adenine failed to induce the phosphorylation of AMPK and ACC following the knockdown of APRT in HUVECs [13].

In the study, we tried to identify whether adenine attenuated ischemia-reperfusion injury in human cardiomyocytes, to evaluate the role of AMPK in the cell-protective effect of adenine, and to find possible substances responsible for signal transduction in the process.

## 2. Materials and Methods

### 2.1. Cell Culture

The H9c2 cells, a cardiomyoblast cell line originally derived from the rat left ventricle, were purchased from the Bioresource Collection and Research Center (BCRC 60096; Hsinchu, Taiwan). The cells were cultured in Dulbecco’s modified Eagle medium (Gibco; Brooklyn, NY, USA) supplemented with 10% fetal bovine serum (Gibco; Brooklyn, NY, USA) and 1% Antibiotic–Antimycotic (Gibco; Brooklyn, NY, USA). Cells were maintained in a humidified atmosphere consisting of 95% air and 5% CO_2_ at 37 °C. In order to prevent differentiation into myocytes by over-confluence, after 2–3 days incubation, H9c2 myoblasts were split at a 1:5 ratio upon reaching 80% confluence. The source of the cells used in this experiment was rats, and no human specimens were used in this study.

### 2.2. Hypoxia–Reoxygenation of H9c2 Cells

After the pretreatment with or without compounds in serum-free media (No FBS) for 3.5 h, simulated ischemia-reperfusion was achieved by culturing the cells in a hypoxia chamber (93% N_2_ + 5% CO_2_ + 1% O_2_) for 21 h. After hypoxia incubation, the cells were exposed to normal oxygen condition (73% N_2_ + 5% CO_2_ + 21% O_2_) for reoxygenation for 6 h [14]. Control cells were cultured in normal conditions. The cells were collected for further analysis.

### 2.3. Cell Number and Volume

We used the ScepterTM handheld automated cell counter (Millipore; Burlington, MA, USA) to determine the cell number, diameter, volume at different adenine concentrations, and reaction time. After the cells were seeded in 3.5 cm dish, the medium was changed every other day (with and without serum), and we added adenine (from Energenesis Biomedical Co., LTD., Taipei, TW.; concentration 100 µM, 200 µM, 400 µM) at a different time than when we treated with hypoxia 21 h and 6 h reoxygenation. The pretreatment dosage of adenine was according to our previous study [13]. Then cells were washed with phosphate-buffered saline (PBS), and trypsin-EDTA was attached to the cells; centrifuged 300 rcf, 3 min; and added 500 µL PBS. We inserted a scepter sensor and analyzed the results with the Scepter Software Pro 2.1 software (Millipore; Burlington, MA, USA).

### 2.4. Cell Viability Assay 

For cell viability experiments, cells were seeded in 96-well cell culture at 2 × 104 cells/well. After 24 h of culture, cells were exposed for 21 h hypoxia–6 h reoxygenation and evaluated using the WST-1 (2-(4-iodophenyl)-3-(4-nitrophenyl)-5-(2,4-disulfophenyl)-2H-tetrazolium) (Roche; Basel, Switzerland). The culture medium was removed, and to the cells 100 µL (1:10 WST-1 and serum free medium) mixture solution was added; they were then incubated for an additional 2 h at 37 °C, protecting the 96 plate from the light. Formazan dye was quantified by measuring its absorbance 450 nm using an ELISA reader [13].

### 2.5. Intracellular ROS/Superoxide Detection

Intracellular ROS levels were measured using a total ROS/Superoxide Detection Kit (Enzo; New York, NY, USA). Regarding the cells seeded in 96-well plates, after 21 h hypoxia and 6 h reoxygenation, the media were deprived and washed once with PBS. We used an ROS/Superoxide Detection reagent and then incubated the media for 1 h, after which we immediately measured Ex and Em wavelengths using standard fluorescein (Ex = 488 nm, Em = 520 nm) and rhodamine (Ex = 550, Em = 610 nm). Then, we substituted the values into the following equation:

Z’ value =1−{[3 × Dsample + 3 × SDcontrol]/[Meansample − Meancontrol]}

### 2.6. Analysis of Glutathione (GSH)

Intracellular GSH was measured using a Glutathione Assay kit (Sigma–Aldrich; St. Louis, MO, USA). Briefly, cells were lysed in 5% 5-Sulfosalicylic Acid (SSA) Solution. Cell lysates centrifuged at 10,000 rcf for 10 min, and supernatants were collected. Supernatants were incubated with working mixture for 5 min. After adding of NADPH, GSH level were monitored at 412 nm using an ELISA reader [14].

### 2.7. Apoptosis and Cell Viability Assay

Annexin V-FITC Apoptosis Detection Kit (Strong Biotech Corporation; Taipei, Taiwan) was utilized to measure H9c2 cell apoptosis and cell viability by following the manufacturer’s instructions. Cells were incubated with 100 μL staining solution (1000 μL Annexin V Binding buffer, 1 μg/mL Annexin V-FITC, 1 μg/mL Propidium Iodide) for 15 min. Cell survival was quantitatively assessed by repeated PI-FITC staining experiments [14].

### 2.8. Quantitative RT-PCR

Relative mRNA levels were examined by quantitative RT-PCR. Total RNA was extracted from cultured H9c2 cells (hearts were homogenized) using TRIzol (Thermo Fisher; Waltham, MA, USA). Then, cDNA was generated using 1 µg total RNA and PrimeScript RT reagent Kit (TaKaRa BIO; Shiga, Japan). Real-time quantitative PCR was performed using the IQ2 SYBR Green Fast qPCR System Master Mix under the following conditions: 95 °C for 2 min followed by 40 cycles at 96 °C for 5 s, 60 °C for 10 s, and 72 °C for 20 s. GAPDH was selected as the reference gene. The PPAR delta and perilipin Ct values were obtained, and the relative fold change in gene expression was calculated after normalizing to beta-actin using the formula [15]:

2 − △Ct

### 2.9. Western Blot Analysis 

The H9c2 cells and left ventricular myocardium lysates were homogenized in cell lysis buffer (PRO-PREPTM; iNtRON Biotechnology; Seongnam, Korea). Lysates were kept on a 4 °C shaker for 5 min for four times and then centrifugated at 16,000 rcf for 20 min at 4 °C; then we collected the supernatants. Protein concentration was determined by BCA Protein Assay Kit (Thermo Fisher; Bannockbum, IL, USA) with bovine serum albumin (BSA) as a standard. Equal amounts of proteins were separated by SDS PAGE using 10% polyacrylamide gel and were transferred onto polyvinylidene difluoride membranes (PVDF, GE Healthcare Life Sciences; Bensalem, PA, USA). The membranes were blocked in 5% nonfat milk for total protein in 0.1% PBST (PBS, 0.01% Tween-20) for 1 h [16].

### 2.10. Antibodies

The antibodies used in this study include β-actin (1:10,000 dilution; Santa Cruz Biotechnology; Santa Cruz, CA, USA), VCP (1:10,000 dilution; GeneTex; Irvine, CA, USA), GRP78 (1:20,000 dilution; GeneTex; Irvine, CA, USA), PDI (1:10,000 dilution; GeneTex; Irvine, CA, USA), ATF4 (1:3000 dilution; GeneTex; Irvine, CA, USA), p-AMPK (1:5000 dilution; Cell Signaling Technology; Boston, MA, USA), Total AMPK (1:10,000 dilution; Cell Signaling Technology; MA, USA), PPARδ (1:10,000 dilution; R&D; Minneapolis, MN, USA), Perilipin (1:10,000 dilution; Abcam; Trumpington, Cambridge, UK), antimouse (1:10,000 dilution; SouthernBiotech; Birmingham, AL, USA), and goat antirabbit (1:10,000 dilution; Santa Cruz Biotechnology; Santa Cruz, CA, USA).

### 2.11. Statistical Analysis

All data were presented as the mean ± standard error of mean (SEM). The statistical significance was evaluated by one-way ANOVA (SigmaPlot 12.0, Systat SigmaStat V3.5.0.54 Software; San Jose, CA, USA). Differences between groups were determined by the Tukey LSD test, with *p* < 0.05 considered statistically significant.

## 3. Results

### 3.1. Protective Effect of Adenine in Hypoxia–Reoxygenation (HR) Cell Model

**Figure 1:**
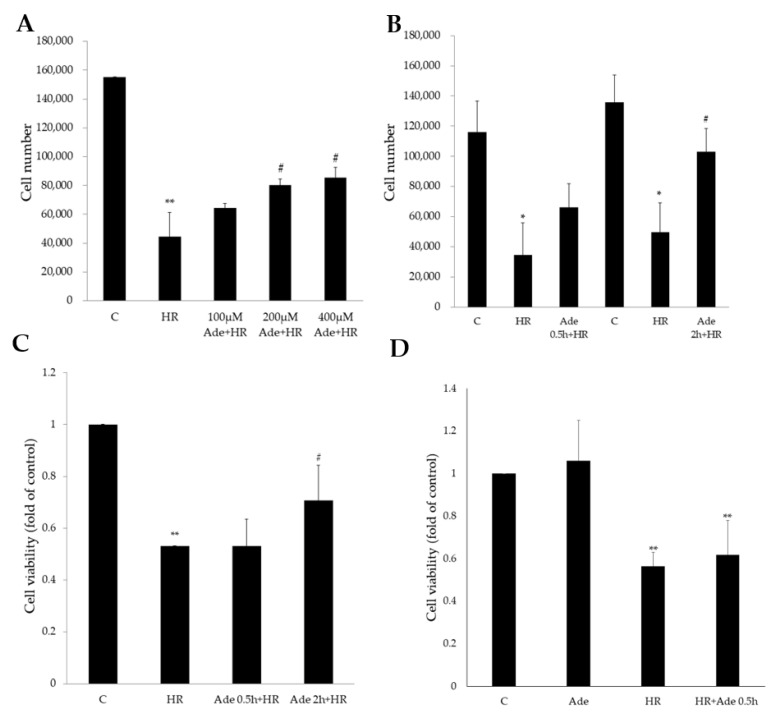
Adenine treatment significantly affected cell numbers and cell viability after hypoxia–reoxygenation (HR). (**A**) HR significantly decreased the cell number in H9c2 cells. Pretreatment with 200 and 400 μM significantly attenuated the HR effects. (**B**) Pretreatment with 200 μM adenine 2 h but not 0.5 h before HR stimulation significantly reversed the HR-decreased cell numbers. (**C**) Pretreatment with adenine significantly reversed HR-decreased cell viability. (**D**) Treatment with 200 μM adenine alone did not increase the cell viability. Treatment with adenine after HR stimulation for 0.5 h did not increase the viability. Each value represents the mean ± SEM; *n* = 6 for each group. * *p* < 0.05 when compared to control group; ** *p* < 0.01 when compared to control; ^#^ *p* < 0.05 when compared to HR group.

H9c2 cells were placed in a hypoxic environment (93% N_2_ + 5% CO_2_ + 1% O_2_) for 21 h and then were returned to a normal, reoxygenation environment (73% N_2_ + 5% CO_2_ + 21% O_2_) for 6 h. The number of H9c2 cells significantly decreased after HR treatment. Pretreatment with 200 μM or 400 μM adenine significantly attenuated HR-induced cell loss in a dose-dependent manner (Figure 1A). The duration of adenine pretreatment also affected the cell survival rate. Pretreatment with 200 μM of adenine for 2 h resulted in more cell protection than pretreatment for 0.5 h in HR injury (Figure 1B). Water-soluble tetrazolium salt (WST)-1 analysis demonstrated that HR significantly decreased cell viability, and pretreatment with adenine for 2 h before HR significantly reduced HR-induced cell death (Figure 1C). On the other hand, treatment of 200 μM adenine after HR stimulation or adenine alone did not increase the cell viability (Figure 1D).

### 3.2. Antioxidant Effect of Adenine

**Figure 2:**
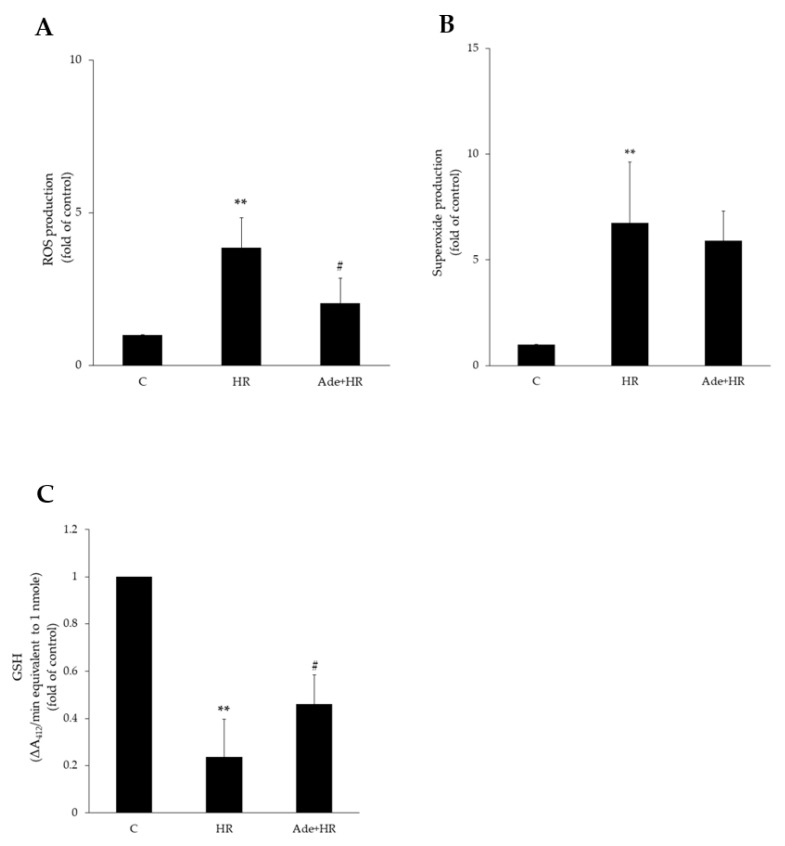
Effects of adenine on hypoxia–reoxygenation (HR)-induced ROS/superoxide in H9c2 cells. (**A**) HR significantly increased ROS production. Pretreatment with 200 μM adenine significantly decreased the productions. (**B**) Pretreatment with adenine reversed the HR-induced superoxide but did not reach statical significance. (**C**) HR significantly decreased GSH production, and pretreated adenine significantly reversed the effects. Each value represents the mean ± SEM; *n* = 6 for each group. ** *p* < 0.01 when compared to control; ^#^ *p* < 0.05 when compared to HR group.

HR treatment increases ROS production and causes free radical accumulation. HR-treated H9c2 cells exhibited significantly increased ROS production compared with the control group. Pretreatment with adenine (200 μM) significantly decreased HR-induced ROS production (Figure 2A) in H9c2 cells. No significant change in superoxide production, however, was noted after adenine pretreatment (Figure 2B). Furthermore, HR also decreased glutathione (GSH), an antioxidant in mitochondria, levels in H9c2 cells. Adenine pretreatment attenuated HR inhibition and reversely increased GSH levels (Figure 2C). These results showed the antioxidant effect of adenine in HR injury.

### 3.3. The Effect of Adenine on Cell Apoptosis after Hypoxia–Reoxygenation

**Figure 3:**
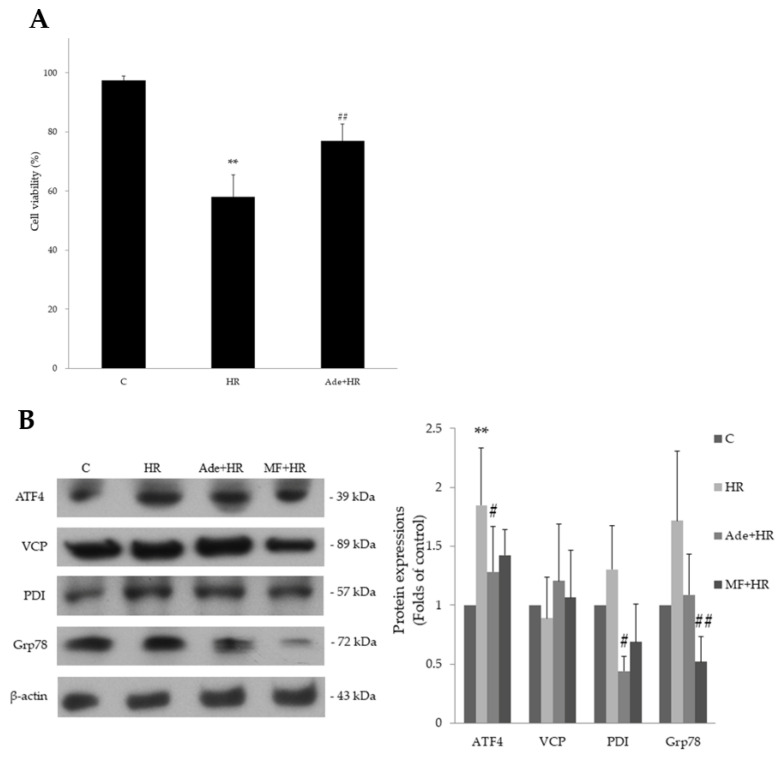
Cell apoptosis detection and endoplasmic reticulum stress protein expression after hypoxia–reoxygenation (HR) treatment and adenine pretreatment. (**A**) Quantification from repeated PI-FITC staining experiments; pretreatment with 200 μM adenine significantly reversed HR-decreased H9c2 cell viability. (**B**) Adenine pretreatment significantly decreased the HR-induced ATF4 and PDI protein expressions in H9c2 cells. Metformin (MF) serves as a comparable control. Each value represents the mean ± SEM; *n* = 6 for each group. ** *p* < 0.01 when compared to control; ^#^ *p* < 0.05 when compared to HR group, ^##^ *p* < 0.01 when compared to HR group.

The protective effect of adenine on HR-induce cell apoptosis was assessed using light phase microscopy, propidium iodide (PI) staining, and fluorescein isothiocyanate (FITC) staining. HR treatment increased cell apoptosis and color density in PI and FITC staining. Pretreatment with 200 μM of adenine significantly decreased cell apoptosis and increased the cell survival rate (Figure 3A). These results suggested that the adenine-treated group had a significantly improved cell survival rate compared with the HR-only group.

### 3.4. The Effect of Adenine on Endoplasmic Reticulum Stress in HR Injury

ER stress is a pathological process in myocardial ischemia-reperfusion injury [17]. The effect of adenine in HR-induced ER stress was studied. HR was shown to induce ER stress and significantly increased the levels of certain ER stress markers, activating transcription factor 4 (ATF4), glucose-regulated protein 78 (GRP78), and protein disulphide isomerase (PDI). Adenine pretreatment significantly attenuated the expression of those proteins in HR injury (Figure 3B). Metformin showed similar effect to adenine. These results indicate that adenine has the ability to attenuate ER stress (Figure S1). This cell-protective effect may be medicated by AMPK.

### 3.5. The Effect of Adenine on AMPK and PPARδ Expression

Activation of PPARδ by PPARδ agonists in human umbilical cord vein cells (HUVECs) was found to attenuate ER stress induced by the plasma from patients with lupus nephritis [18]. Activation of AMPK was also shown to inhibit ER stress in previous studies [19,20]. We attempted to identify the relationship among HR, ER stress, PPARδ, and AMPK in this study.

**Figure 4:**
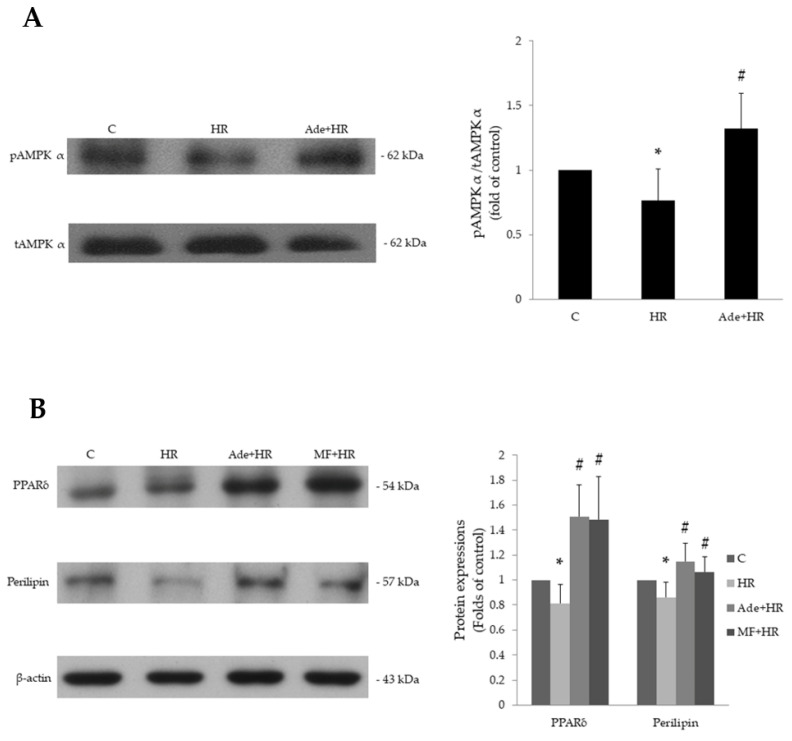
Adenine effects on AMPK and PPARδ expressions after hypoxia–reoxygenation (HR). (**A**) Pretreatment with 200 μM adenine significantly reversed the HR-decreased ratio of phosphorylation AMPK and total AMPK protein expressions. (**B**) HR treatment significantly decreased PPARδ and downstream perilipin protein expressions, and pretreatment with adenine or metformin significantly increased PPARδ and perilipin expressions after HR stimulation, respectively. Each value represents the mean ± SEM; *n* = 6 for each group. * *p* < 0.05 when compared to control; ^#^ *p* < 0.05 when compared to HR group.

HR was shown to significantly decrease the ratio of phosphorylated AMPK to total AMPK in H9c2 cells. Pretreatment with adenine significantly reversed this inhibition and elevated phosphorylated AMPK levels (Figure 4A). The expression of PPARδ and a downstream signaling substance, perilipin, was also attenuated by HR. Pretreatment with adenine significantly elevated HR-inhibited PPARδ and perilipin expression. Pretreatment with metformin, a well-documented AMPK activator, also reversed the HR-induced inhibition of PPARδ and perilipin expression (Figure 4B). These results indicate that phosphorylation of AMPK is inhibited in HR injury (Figure S2). After adenine administration, AMPK may be activated first by adenine and subsequently increases PPARδ and downstream perilipin expression.

## 4. Discussion

This study showed that hypoxia–reoxygenation (HR) injury in cardiomyocytes induced ROS production, cell apoptosis, and ER stress, which may be attenuated by pretreatment with adenine. The inhibitory ability of adenine may be mediated through AMPK and PPARδ signaling.

The endoplasmic reticulum (ER) serves several essential cellular functions including protein synthesis, protein folding, protein translocation, calcium homoeostasis, and lipid biosynthesis. Pathological conditions disrupting ER homoeostasis may lead to accumulation of misfolded and unfolded proteins, a condition referred to as ER stress. ER stress triggers the unfolded protein response (UPR) to restore ER homoeostasis through activating several signal transduction pathways. Prolonged ER stress will lead to cell dysfunction and apoptosis [21].

ER stress plays a major role in cardiac myopathies. Activation of AMPK was shown to inhibit ER stress in previous studies [19,20]. Ezetimibe, a cholesterol absorption inhibitor, was shown to reduce IR-induced oxidative stress and UPR pathway activation through activation of AMPK [22]. Pang et al. proved that CAMKKβ/AMPK/mTOR-dependent signaling pathway was critical for inhibiting ER stress in cardiomyocytes [23]. A mitochondrial aldehyde dehydrogenase-2 study indicated the critical role of mitochondrial function in the cardiomyopathy [24]. These studies confirmed the relationship between mitochondria and pathogenesis of heart diseases. Adenine was a novel AMPK activator and key compound in mitochondria. In our study, we treated H9c2 cells with adenine, which inhibited ER stress and increased the cell viability after HR stimulation. The results demonstrated the beneficial effects of adenine in inhibiting the cardiomyopathy.

Peroxisome proliferator-activated receptor delta (PPARδ), one of three members of the PPAR group in the nuclear receptor superfamily, is a ligand-activated transcription factor. PPARδ regulates important cellular metabolic functions that contribute to maintaining energy balance [25]. Activation of PPARδ by PPARδ agonists in human umbilical cord vein cells (HUVECs) attenuated ER stress induced by the plasma from patients with lupus nephritis [18]. Perilipins, a kind of downstream signaling protein following PPARδ activation [26], were shown to protect against lipotoxicity and reduce endoplasmic reticulum stress in pancreatic β-cells [27,28].

The relationship between PPARδ and AMPK is still not clear enough. Activation of PPARδ was shown to increase AMPK phosphorylation and decrease ER stress-induced oxidative stress [29]. In this study, the expression of PPARδ and perilipin were increased by adenine and metformin after HR injury. The expression of several ER stress markers, such as activating transcription factor 4 (ATF4), glucose-regulated protein 78 (GRP78), and protein disulphide isomerase (PDI), were also inhibited by adenine and metformin. According to the AMPK-activating ability of metformin, adenine may activate AMPK first and sequentially activate PPARδ signal pathway to attenuate ER stress in HR injury. Further studies are needed to identify the underlying mechanism in detail.

## 5. Conclusions

This study was the first to investigate the relationship between adenine pretreatment time and HR-induced ER stress in H9c2 cells. These results indicated that effects of adenine related to AMPK and PPAR delta signaling. Further animal experiments and human clinical studies will need to verify the results of this study [30] so we can translate adenine into clinical treatment of myocardial hypoxia and reoxygenation. In the future, there is an opportunity to develop adenine as a preventive drug that is given before the reoxygenation procedure in myocardial infarction patients.

## Data Availability

The data presented in this study are available on request from the corresponding author.

## Supplementary Materials

The following are available online at https://www.mdpi.com/article/10.3390/life11121408/s1, Figure S1: Original western blot figures ER (a–t), Figure S2: Original western blot figures PPARPeri (a–l).
