# Supplementary material for: The Cell Protective Effect of Adenine on Hypoxia–Reoxygenation Injury through PPAR Delta Activation
Source: Life (Basel). 2021 Dec 16;11(12):1408. doi: 10.3390/life11121408 (PMC8703696; doi:10.3390/life11121408)
Supplement: Supplementary file 1 [file life-11-01408-s001.zip › life-1470814-supplementary.pdf]

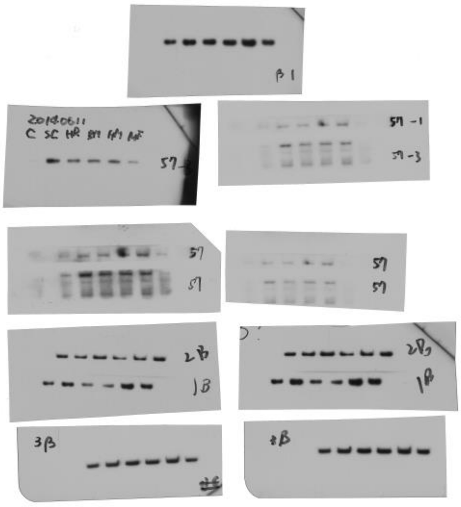

(a)

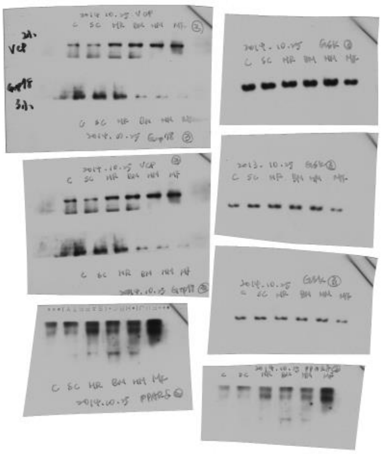

(b)

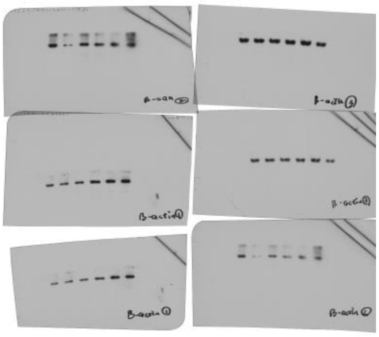

(c)

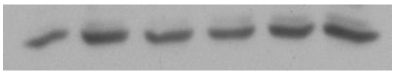

(d)

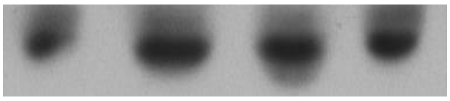

(e)

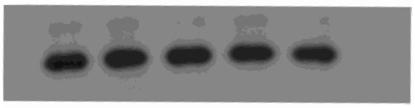

(f)

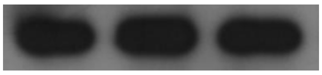

(g)

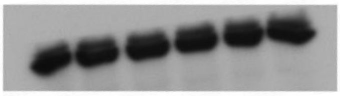

(h)

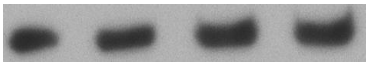

(i)

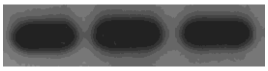

(j)

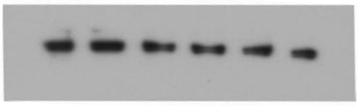

(k)

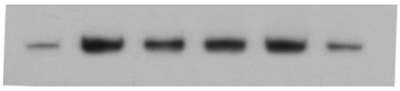

(l)

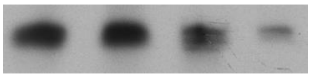

(m)

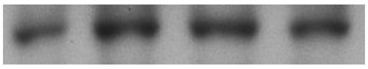

(n)

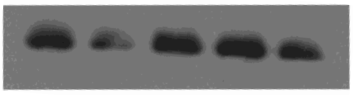

(o)

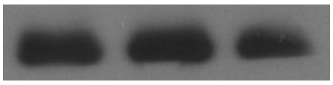

(p)

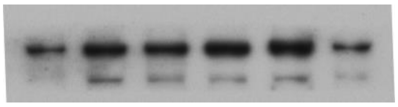

(q)

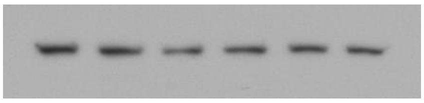

(r)

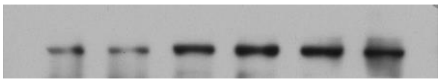

(s)

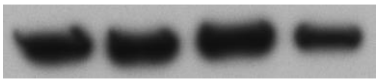

(t)

Figure S1. Original western blot figures ER **(a-t).**

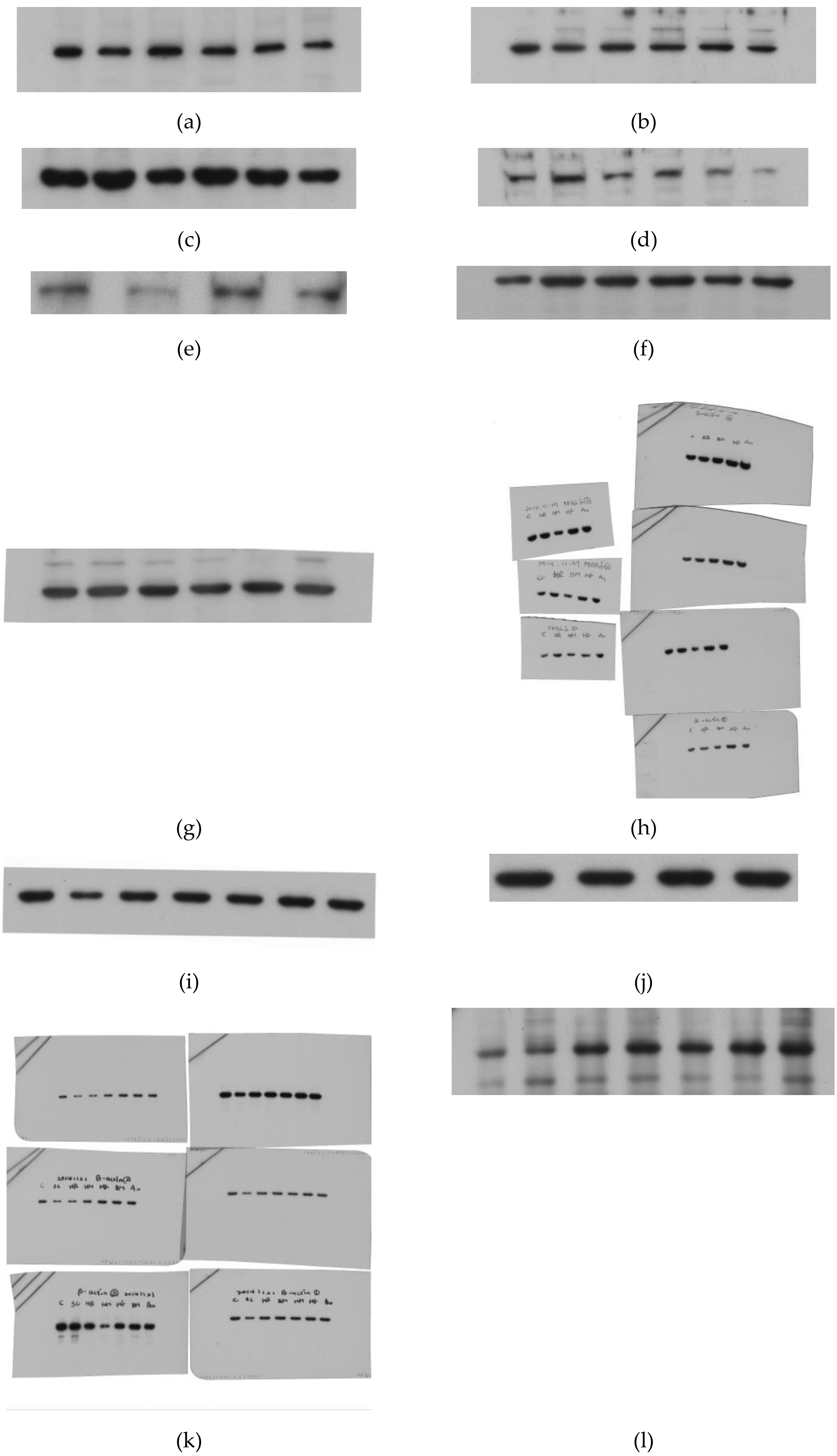

Figure S2. Original western blot figures PPARPeri **(a-l).**
